# Are Young Men Getting the Message? Age Differences in Suicide Prevention Literacy among Male Construction Workers

**DOI:** 10.3390/ijerph16030475

**Published:** 2019-02-06

**Authors:** Tania L. King, Philip J. Batterham, Helen Lingard, Jorgen Gullestrup, Chris Lockwood, Samuel B. Harvey, Brian Kelly, Anthony D. LaMontagne, Allison Milner

**Affiliations:** 1 Centre for Health Equity, School of Population and Global Health, University of Melbourne, Melbourne 3010, Australia; allison.milner@unimelb.edu.au; 2 Centre for Mental Health Research, Research School of Population Health, Australian National University, Canberra 2601, Australia; Philip.Batterham@anu.edu.au; 3 Construction Work Health and Safety Research @ RMIT, School of Property, Construction and Project Management, RMIT University, Melbourne 3000, Australia; helen.lingard@rmit.edu.au; 4 MATES in Construction, Spring Hill 4000, Australia; jorgen@micqld.org.au (J.G.); clockwood@micaus.org.au (C.L.); 5 Black Dog Institute, Faculty of Medicine, University of New South Wales, Sydney 2052, Australia; s.harvey@unsw.edu.au; 6 School of Medicine and Public Health, University of Newcastle, Newcastle 2308, Australia; brian.kelly@newcastle.edu.au; 7 Work, Health and Wellbeing Unit, Centre for Population Health Research, School of Health & Social Development, Deakin University, Geelong 3217, Australia; tony.lamontagne@deakin.edu.au

**Keywords:** mental health, suicide, age, men, construction workers, beliefs, intervention, workplace

## Abstract

Suicide is a leading cause of death among young men. Help-seeking is known to be poor among this group, and little is known about what interventions are most successful in improving suicide prevention literacy among young men. This research aims to examine: (1) age differences in beliefs related to suicide prevention literacy and attitudes to the workplace in addressing mental health among male construction workers; (2) age differences in response to a workplace suicide prevention program. Pre- and post-training survey data of 19,917 male respondents were obtained from a workplace training program database. Linear regression models and predictive margins were computed. Mean differences in baseline beliefs, and belief change were obtained for age groups, and by occupation. Young men demonstrated poorer baseline suicide prevention literacy but were more likely to consider that mental health is a workplace health and safety issue. There was also evidence that young men employed in manual occupations had poorer suicide prevention literacy than older men, and young men employed in professional/managerial roles. The youngest respondents demonstrated the greatest intervention-associated change (higher scores indicating more favourable belief change) to *People considering suicide often send out warning signs* (predicted mean belief change 0.47, 95% CI 0.43, 0.50 for those aged 15–24 years compared to 0.38, 95% CI 0.36, 0.41 for men aged 45 years and over), and to *The construction industry must do something to reduce suicide rates* (predicted mean belief change 0.17, 95% CI 0.15, 0.20 for those aged 15–24 years compared to 0.12, 95% CI 0.10, 0.14 among men aged 45 years and over). Results indicate that while suicide prevention literacy may be lower among young men, this group show amenability to changing beliefs. There were some indications that young men have a greater propensity to regard the workplace as having a role in reducing suicide rates and addressing mental health, highlighting opportunity for workplace interventions.

## 1. Introduction

Suicide and intentional self-harm are major causes of morbidity and premature death worldwide, and are of significant public health importance. Rates of suicide and self-harm are known to vary by population characteristics, particularly by age and gender. Suicide is a leading cause of death for young people both internationally [1,2], and within Australia [3]. In 2016, over a third (35.4%) of deaths among those aged 15–24 years were due to suicide, and more than one quarter of deaths among those aged 25–34 years were attributed to suicide [3]. In Australia [3], as in many parts of the world [1,4], male adolescents and young men are more likely to die by suicide than young females.

The suicide rate among young males aged 15–34 years in Australia peaked in the mid-1990s, and has declined since then [5]; however, clear socioeconomic differences remain, with young men in areas of high disadvantage at greater risk of suicide than those in more advantaged areas [6]. This is consistent with research showing that low educational attainment and low socioeconomic status are risk factors for self-harm and suicide in young people [4]. It is also known that occupation (also commonly considered a marker of socioeconomic position) can be a risk factor for suicide [7]. In particular, research suggests that those in the highest occupational skill level group have lower rates of suicide, while those employed in the lowest skill level such as labourers, cleaners, or plant operators have higher rates of suicide [7]. Australian research also highlights that those employed in manual occupations, including construction workers and tradespeople are at higher risk of suicide than the rest of the working population [8]. There is some evidence to suggest that young men in the construction industry have higher suicide rates than older men [9].

Possessing knowledge and understanding of mental health that may assist in the recognition of symptoms, management and prevention is defined as “mental health literacy” [10]. Poor mental health literacy is known to impede early symptom recognition in the self, as well as in others, and this in turn, may delay or stymie help-seeking behaviour [10]. Mental health literacy is known to vary across population groups, and males have been identified as being less adept than females at correctly recognising symptoms of mental illness [11,12]. Other research has shown that recognition of mental health symptoms varies across age groups, with evidence that younger groups have better mental health literacy than older groups [13]. Suicide prevention literacy is one element of mental health literacy, and is defined as an individual’s knowledge about: suicidal behaviours or self-harm in terms of risks and protective factors; the forms of help available; and where and how to access help [14]. Males are known to have poorer suicide prevention literacy than females, and there is evidence that younger groups have better suicide prevention literacy than older groups in the general population [15]. Little is known, however, about age differences in suicide prevention literacy within the construction industry.

While mental health literacy is key to help-seeking behaviours, there is evidence that a large proportion of those with a mental health disorder do not seek formal help [16] or receive professional treatment [17]. Encouraging help-seeking from formal treatment services has been a focus of many programs; however, the extent to which this is effective is unknown. Help-seeking is a complex decision-making process [18], and there are salient age differences in approaches to treatment and help-seeking [19]. Most notably, young people are more likely to endorse more informal sources of help for mental illness, while older adults regard few treatment sources (either formal or informal) as helpful [19]. Furthermore, there is evidence that lay sources of help such as family and friends are preferred in times of stress and strain [16]. Together with building knowledge and awareness of mental health and suicide, fostering effective support among proximal others is recognised as a key support strategy [16].

Given the observed higher risk of self-harm and suicide among younger males, together with evidence of greater suicide risks within the construction industry, examining suicide prevention literacy among this group is critical. Identifying gaps or points of improvement in suicide prevention literacy is critical to the development of suicide prevention strategies. The workplace may be an important, but under-utilised setting for both examining suicide prevention literacy, and intervening to improve mental health literacy and prevent suicide among young men. “MATES in Construction” (MATES) was established as an industry-based, workplace-focused program that seeks to prevent suicide [20]. Described in detail elsewhere [21,22], MATES aims to: raise awareness about suicide in the workplace; reduce stigma associated with mental health and help-seeking; facilitate and support help-seeking; ensure the appropriateness and viability of help provided. General Awareness Training (GAT) is a central component of MATES [22], and involves a one-hour training session in which the principal aim is to engage and activate construction workers in suicide prevention. MATES has systematically collected pre–post evaluation information from all participants in the program since its establishment.

Using GAT evaluation information, we aimed to examine age differences in suicide prevention literacy among construction workers, as well as in attitudes to the workplace in addressing mental health. This information is critical to informing intervention design and strategy development. A secondary aim was to compare change in suicide beliefs across age groups in response to the intervention. Based on evidence that younger men have better mental health literacy [13] and greater suicide literacy [15] than older men, we hypothesised that young men in our sample would have better suicide prevention literacy, be more receptive to the role of the workplace in preventing suicide, and show greater change in beliefs. Our final aim was to examine age differences in beliefs across occupational groups. Based on existing evidence on the impact of educational attainment, we hypothesised that young people employed in professional and managerial occupations would show better suicide literacy than those in more manual occupations.

## 2. Materials and Methods

### 2.1. Data Source

Data were from a MATES collected database of pre- and post-GAT evaluation results between 2016 and 2018. We chose these three years as the GAT questionnaire was consistent throughout this time. Data were collected using paper surveys, with the baseline survey conducted before the training, and the follow-up conducted immediately afterwards. Importantly, data were collected by the program provider with minimal burden on participants for the purposes of on-going/general evaluation, rather than in the context of a purpose-designed evaluation research study.

### 2.2. Participants

Public and private sector construction sites across four states of Australia (New South Wales (NSW), Western Australia, South Australia, and Queensland) were recruited to participate in MATES in Construction. All workers at these sites were invited to participate in GAT. Workers completing GAT were provided with a white MATES sticker to wear on their hard hat. For a job site to be “MATES compliant”, all workers on a given worksite must be exposed to GAT, and an 80% training level must be maintained irrespective of staff turnover, and inclusive of sub-contracted workers, who are highly transient.

### 2.3. Ethics

This research was approved by the University of Melbourne Human Ethics Committee #1750927.

### 2.4. Evaluation Design

This evaluation involved an uncontrolled pre–post-test design, with pre–post data linked within individuals.

### 2.5. GAT Intervention

GAT is a one-hour training session provided to all construction workers on sites recruited to the MATES program. It is provided by MATES staff, including field officers and management, either as a stand-alone session, or as a component of the Life Skills Toolbox, a training program for apprentices. On average, each training session contained 48 participants.

This universal intervention aims to increase suicide prevention literacy and position it as a workplace health and safety issue. In doing this, it aims to improve knowledge regarding suicide warning signs, and encourage workers to offer support to co-workers who display warning signs of possible increased suicide risk.

### 2.6. Measures

Prior to GAT, participants completed a short questionnaire. This sought basic demographic information including gender, occupation, age, postcode of residence, state of training, as well as responses to a set of statements examining suicide prevention literacy (refer to Supplementary Material for survey).

#### 2.6.1. Exposure

We were interested in age differences in suicide prevention literacy and attitudes to the workplace in preventing suicide, so age was our key “exposure” of interest, measured by a categorical variable: 15–24 years, 25–34 years, 35–44 years, and 45 years and older.

#### 2.6.2. Outcomes

At both pre- and post-tests, participants indicated agreement (on a 5-point Likert scale, from strongly agree (1) to strongly disagree (5)) on four statements that assessed their beliefs and awareness regarding suicide prevention, and the role of the workplace in preventing suicide. Details on the outcome items have been documented elsewhere [23], and were based on field trials of items drawn from “ten myths about suicide” documented by the World Health Organisation [24]. We considered combining the four items into a single unidimensional measure; however, the internal consistency, as measured by Cronbach’s alpha, was insufficient (α = 0.32). There are also conceptual motivations for examining these separately: two of the items reflect “suicide prevention literacy”, and the other two items reflect “attitudes to the workplace in preventing suicide”. Given these motivations, together with evidence that there are substantial differences in response to these measures [23], we chose to examine them separately.

##### Suicide Prevention Literacy

These reflected beliefs about suicide prevention literacy and were: “*Talking about suicide can cause suicide*”; “*People considering suicide often send out warning signs or invitations*” (herein referred to as “*People considering suicide often send out warning signs*”). To assess suicide prevention literacy, we used pre-test scores on these two items. 

##### Attitudes to the Workplace in Preventing Suicide

Two pre-test items were used to assess attitudes to the workplace in preventing suicide. These were: “*Poor mental health is a workplace health and safety issue*” and “*The construction industry must do something to reduce suicide rates*”.

##### Change in Beliefs 

To examine change, a “change in belief” variable was created from pre- and post-test measures and was obtained by subtracting the pre-test score from the post-test score.

### 2.7. Covariates

We adjusted for available covariates in analytic models. MATES has been present in different states for different periods of time; therefore, we also adjusted for state of training (NSW, Queensland, Western Australia, and South Australia). We also adjusted for year of training (2016, 2017, and 2018), as well as training session (continuous variable). Some sets of analysis also made adjustment for occupation, using an eight-category, one-digit skill level classification based on the Australian Standard Classification of Occupations. As there were no respondents in two of these categories, the analysis contained six occupational categories. We also note that apprentices, while technically not classified in the occupational grouping, were classified as “labourers” (lowest-skill group). Sensitivity analysis also made adjustment for previous experience of suicide, measured by an item in which respondents were asked whether they had known anyone who had died by, or attempted suicide (Yes/No).

### 2.8. Analytic Sample

Analysis was restricted to those completing their first GAT (we excluded n = 4214 completing their second or subsequent training session). Of the 22,206 workers in the eligible sample, n = 1456 were excluded due to missing beliefs, and n = 1289 were dropped from analysis due to missing age information. After exclusion of missing data (10.3%), the resultant sample was n = 19,917. Given the considerable missing data on occupation (35%), it was not included in the main analysis.

### 2.9. Statistical Analysis

The statistical analysis was conducted using the software Stata, version 15 [25].

To assist with the interpretation of results, we recoded the belief variables so that the desirable responses for all beliefs ran in the same direction, with higher scores indicating desirable belief endorsement.

We fitted models using the xtmixed command in Stata. Models were adjusted for state, year of training and clustering by the training session (Model a). Previous work has shown that the majority of respondents have known someone who has attempted/died by suicide [23]. Given that those in the youngest age group (15–24 years) were slightly less likely to report this (70.4%, compared to 74–77% among the older groups), we also examined whether controlling for previous experience of suicide (knowing someone who had died by, or attempted suicide) affected beliefs (Model b). Additional models also included occupation as a covariate (Model c). The margins postestimation command was used to calculate the predicted mean beliefs (adjusted) based on the entire analytic sample. 

We also ran a similar set of models for belief change. As for baseline beliefs, Model a contained estimates adjusted for state, year of training and clustering by training session. Model b adjusted for knowledge of someone who had attempted/died by suicide, and Model c adjusted for occupational skill level.

## 3. Results

### 3.1. Sample Descriptives

More than half of respondents were aged 25–44 years, and respondents from Queensland constituted almost half of the sample, with NSW being the next largest group. More than two-thirds of data collection occurred in 2017 (see Table 1). Technicians and trades-workers were the largest single occupational groups, accounting for more than two-fifths of the sample. More than one quarter of those providing occupational information were labourers. There were a similar number of machinery operators and managers, but only a small number of professionals and clerical/administrative workers.

### 3.2. Baseline Beliefs

On both measures of suicide prevention literacy, young men reported poorer (lower) beliefs. *People considering suicide often send out warning signs* evoked least desirable beliefs for all age groups, but particularly for those aged 15–24 (predicted mean belief 3.15, 95% confidence interval (CI) 3.11, 3.19), compared to all other age groups, such as those aged over 45 years (predicted mean belief 3.41, 95% CI 3.38, 3.44) (see Table 2). For *Talking about suicide can cause suicide*, young men reported least desirable beliefs (predicted mean belief 3.46, 95% CI 3.43, 3.50), compared to all other age groups, and those aged 35–44 had the most desirable beliefs (predicted mean belief 3.65, 95% CI 3.62, 3.68).

The age differences in baseline beliefs were also apparent when comparing the beta coefficients arising from the linear regression results (15–24 year olds were the reference category, and the coefficients indicate the mean difference in beliefs across age groups). For Model a, the beta coefficients for the mean differences for both suicide literacy beliefs indicate more desirable beliefs for all age groups, compared to those aged 15–24 years. The inclusion of a variable capturing knowledge of someone who had attempted/died by suicide (Model b) and occupation (Model c) did not substantially change estimates for suicide prevention literacy.

While the age groups did not differ in terms of beliefs regarding the role of the construction industry in reducing suicide rates (Table 2), the two younger age groups (15–24 and 25–34 years) showed more desirable beliefs regarding the role of the workplace (*Poor mental health is a workplace health and safety issue*). For those aged 15–24 years, the predicted mean score in relation to this belief was 4.21, (95% CI 4.18, 4.23), and for those aged 25–34 years, the predicted mean was 4.23, (95% CI 4.21, 4.25), compared to 4.12, (95%CI 4.10, 4.15) and 4.09, (95% CI 4.07, 4.11) for those aged 35–44 years and 45 years and over, respectively. The linear regression results for mean differences in attitudes to the workplace in preventing suicide also evinced these associations (Table 2): estimates remained largely unchanged with the inclusion of a variable measuring knowledge of someone who had died by/attempted suicide (Model b) and occupation (Model c).

### 3.3. Occupational Differences

**Figure 1:**
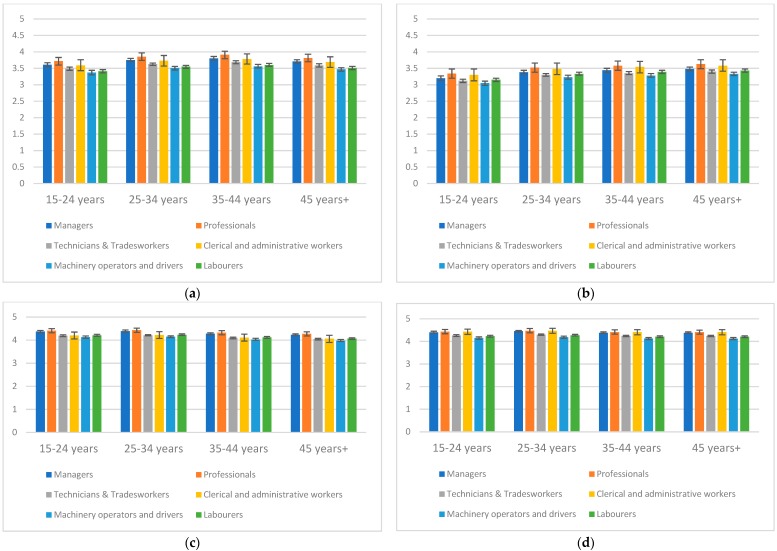
Occupational differences in baseline beliefs by age group. (**a**) Talking about suicide can cause suicide; (**b**) people considering suicide often send out warning signs; (**c**) poor mental health is a workplace health and safety issue; (**d**) the construction industry must do something to reduce suicide rates.

We also examined occupational skill level differences within each age group, noting that this information was not available for all participants (see Figure 1a–d, as well as Table S1 in Supplementary Materials).

For *Talking about suicide can cause suicide* and *People considering suicide often send out warning signs*, differences in beliefs were patterned by occupational skill level. In particular, those working in manual roles (technicians and trades-workers, labourers, machinery operators and drivers) had the poorest beliefs within each age group, but this was particularly observable among 15–24 year olds. For example, for *Talking about suicide can cause suicide*, the predicted mean beliefs for 15-24 year old technicians and trades-workers (predicted mean belief 3.49, 95% CI 3.44, 3.54), labourers (predicted mean belief 3.41, 95% CI 3.36, 3.46), machinery operators and drivers (predicted mean belief 3.37, 95% CI 3.30, 3.44) were notably poorer than for other groups such as professionals (predicted mean belief 3.72, 95% CI 3.60, 3.83). We attempted to test for age interactions, but cell sizes were insufficient to enable this.

The main analysis reported in Table 2 showed that men in the youngest age group in the sample reported most desirable beliefs in relation to *Poor mental health is a workplace health and safety issue*. The occupational analysis reinforced this, but also showed that this varied across occupation. While those aged 15–24 years employed as technicians and trades-workers, labourers, machinery operators and drivers showed more desirable beliefs (predicted mean belief 4.19, 95% CI 4.15, 4.23; predicted mean belief 4.21 95% CI 4.17, 4.25; predicted mean belief 4.13, 95% CI 4.08, 4.18 respectively) than men aged 45 years and over in the same occupations (predicted mean belief 4.05, 95% CI 4.01, 4.08; predicted mean belief 4.07, 95% CI 4.03, 4.10; predicted mean belief 3.98, 95% CI 3.94, 4.03 respectively), these beliefs were poorer than those of professionals aged 15–24 years (predicted mean belief 4.41, 95% CI 4.32, 4.50). 

### 3.4. Belief Change

We also investigated change in beliefs by comparing differences in pre- and post-beliefs (Table 3; higher positive scores indicating greater desirable belief change). There were no age differences in change for *Talking about suicide can cause suicide*. There were similarly no differences across age groups for change in response to *Poor mental health is a workplace health and safety issue*.

*People considering suicide often send out warning signs* produced greatest change in beliefs for all age groups; however, the youngest age groups demonstrated the greatest change. This was apparent in both the predicted mean belief change, as well as the beta coefficients measuring mean difference in belief change arising from the linear regression models (for the beta coefficients for mean difference in belief change, a negative coefficient indicates that the change was less than that of the reference category, in this case 15–24 year olds). Compared to 15–24 year olds, those aged 35–44 years (β −0.07, 95% CI −0.11, −0.03), and those aged 45 years and over (β −0.08, 95% CI −0.13, −0.04) showed the least desirable belief change. Estimates did not change, depending on whether respondents reported knowing someone who had attempted/died by suicide; however, the inclusion of occupation in this model strengthened associations between age and belief change.

For *The construction industry must do something to reduce suicide rates*, those aged 15–24 years showed a greater (desirable) change in beliefs than men aged 35–44 years (β −0.04, 95% CI −0.07, −0.01), and men aged 45 years and over (β −0.06, 95% CI −0.09, −0.03). The inclusion of knowledge of someone who had attempted/died by suicide and occupational skill level led to a negligible change in estimates.

## 4. Discussion

These results present important evidence that baseline suicide prevention literacy, and attitudes to the workplace in addressing suicide and mental health differ by age group. Contrary to our hypothesis that young men would display better suicide prevention literacy, we found evidence that young men had poorer suicide prevention literacy than older age groups. We also found evidence that young men were more likely to endorse the role of the workplace in addressing mental health. These results were not attenuated by adjustment for having known someone who had attempted/died by suicide, or by occupational skill level.

With respect to intervention-associated change, age differences for some items were also apparent, with the young men in the sample showing greater amenability to belief change for some beliefs, particularly for *People considering suicide often send out warning signs*, and *The construction industry must do something to reduce suicide rates*, where belief changes were greater (desirable) for 15–24 year olds, compared to those aged 35–44 years and 45 years and older.

According to standard occupational classifications, the construction workforce can be divided into two groups of workers: those who work in manual, non-managerial construction work and those who work predominantly in an office environment. Occupational patterning aligning with these distinctions was observed across all age groups, with those in the manual/non-managerial groups (labourers, technicians and trades workers and machinery operators/drivers) showing poorer suicide literacy and lower endorsement of the role of the workplace/industry in addressing mental health and suicide than those in more managerial/office-based roles (managers and professionals). These differences may suggest differences in worksite culture between those employed in office-based roles, and those employed in manual/non-managerial roles. If this is the case, the delivery of workplace suicide prevention programs is critical to ameliorate these worksite cultural differences.

The lower suicide prevention literacy of young manual workers was particularly salient, with those employed as labourers, technicians and trades-workers, and machinery operators and drivers showing lower endorsement of *People considering suicide often send out warning signs* and higher (undesirable) endorsement of *Talking about suicide can cause suicide*. These occupational differences highlight vulnerabilities among young manual workers, suggesting that they should be a focus of suicide prevention programs and initiatives.

We note that the differences reported here are small, and may have negligible impact on an individual’s mental health or suicide risk. However, we also note that small individual shifts in risks or scores may still be important at a population level, serving to shift the proportion of individuals at the high-risk tail end of the population [26].

These results contrast with some previous work. Age differences in mental health and suicide literacy have previously been reported, and have indicated that younger groups have better mental health literacy [13] and better suicide prevention literacy [15] than older groups. We note, however, that we are unaware of previous work on suicide prevention literacy within the construction industry. It is possible that the slightly better suicide prevention literacy observed in the older groups in the construction setting reflects greater exposure to workplace suicide prevention programs such as MATES. Future analysis will explore this by examining the effect of length of time in the industry.

The other substantive results arising from this research are those showing differences in baseline attitudes to the workplace—those aged 15–24 years (and those aged 25–34 years) showed greater endorsement of the workplace, as having some responsibility in addressing mental health than those aged 35–44 years and 45 years and over. This attitude to the workplace is noteworthy and suggests potential to strengthen mental health in this vulnerable group. It is known that young males are reticent to seek help for mental health problems from professional sources [27], so their affirmation of the workplace as having a role in addressing mental health is important. Furthermore, given the lower suicide prevention literacy observed in this group, this result is important because it suggests that younger groups may be receptive to workplace mental health and suicide education and prevention programs.

The fact that older workers were less likely to regard the workplace as having some responsibility to address mental health is also of importance, because suicide is the second leading cause of death among Australian men aged 45–54 years [3]. It is unclear whether this age difference is associated with a natural belief trajectory (that workers increasingly abjure this belief as they spend more time in the industry) or a cohort effect (that younger men generally have a belief in the wider workplace responsibility in addressing mental health). It is in fact possible that this reflects generational change in expectations about workplace health and safety, with recognition among young men that this is now broader than control of physical safety risks. Continued evaluation over time is necessary to understand this.

It is possible that the reduced endorsement of the workplace as having some responsibility to address mental health that we observed among males aged 35 and older may reflect the enactment of masculine norms (which similarly may be either a cohort effect or an industry effect). The construction industry is known to be a key setting in which hegemonic masculinities remain inveterate [28]. Masculine ideals are founded on toughness, control, autonomy and competence [29]: help-seeking and admitting a need for help violate these standards. The stronger endorsement of the role of the workplace in relation to mental health observed among the younger males may be interpreted positively, and may suggest a reduced adherence to damaging masculine norms in relation to being able to cope. The potential that masculine norms underpin the reduced endorsement of beliefs regarding the responsibility of the workplace that was observed among males aged 35 years and over, points to opportunities for further research to direct interventions to address norms that may inhibit help-seeking and help-offering opportunities among population groups at risk of suicide.

We note some limitations of this analysis. Firstly, we acknowledge that there was a high level of missing data on occupation. For this reason, we present models with and without adjustment for occupation. In relation to occupation, we also note that some of the occupational categories, notably professionals and managers, often denote occupations such as engineers that require 3–4 years of university training. Thus, the number of professionals and managers in the younger age groups was relatively small.

As noted elsewhere [23], GAT was implemented for the purposes of on-going/general evaluation, rather than as a purpose-designed evaluation research study. Additionally, GAT is delivered by many trainers across Australia, and while its delivery is standardised, variations are highly possible. We attempted to control for this by adjusting for clustering by training session; however, this may have inadequately controlled for trainer variation.

It is also possible that our results were biased by our inability to control for prior mental health status. Further, it is commonly assumed that the provision of information about stress and mental health issues improves mental health outcomes through several mechanisms, including facilitating help-seeking and support (and enabling earlier treatment), introducing corrective information, and enabling greater awareness of emotional range; however, some have queried these assumptions [30]. Some researchers have further argued that mental health education alone may not be beneficial, and may in fact present risks [30,31]. Importantly too, the post-test information was collected immediately after the intervention; we therefore have no way of knowing whether the intervention exerted a persistent effect or not. Longitudinal studies are needed to ascertain the enduring effects of suicide prevention programs. As an additional limitation, the intervention was delivered to the whole sample, with no control or comparison group, thus limiting causal inference.

There are several strengths of this analysis. A key strength is that this work substantially adds to the body of knowledge regarding suicide literacy among young construction workers—a highly under-researched population. We also note that our results remained robust, whether occupation or knowing someone who had attempted/died by suicide was included in the model. We used a large dataset, which provides a strong basis for statistical inference.

## 5. Conclusions

We present results showing age differences in suicide and mental health understanding, as well as differences in amenability to change following a workplace intervention. We found evidence that young males have poorer suicide prevention literacy than older groups; however, there is evidence that they are amenable to belief change, and also report greater endorsement of the belief that the workplace has a role in addressing mental health than other groups. This suggests receptiveness to workplace suicide prevention intervention, and points to opportunities to implement workplace programs to improve suicide prevention literacy among young men.

## Figures and Tables

**Table 1:** Sample characteristics (total n = 19,917).

Variable	n	%
Age at training		
15–24 years	2961	14.9
25–34 years	6297	31.6
35–44 years	4769	23.9
45+ years	5890	29.6
State of training		
New South Wales	5554	27.9
Queensland	9024	45.3
Western Australia	1927	9.7
South Australia	3412	17.1
Year of training		
2016	4787	24.0
2017	13,650	68.5
2018	1480	7.4
Knowledge of someone who attempted/died by suicide *		
Yes	14,599	74.5
No	4987	25.5
Occupational category **		
Managers	1952	15.1
Professionals	202	1.6
Technicians & Trades-workers	5466	42.3
Clerical and administrative workers	113	0.9
Machinery operators and drivers	1850	14.3
Labourers	3335	25.8

* n = 19,586 due to missing data. ** n = 12,918 due to missing data.

**Table 2:** Predicted mean baseline beliefs and mean differences in baseline beliefs.

Belief	Age Group	Predicted Means (Model a)	Mean Difference in Baseline Beliefs, β Coefficient (95% CI)		
			Model a	Model b	Model c
Talking about suicide can cause suicide	15–24 years	3.46(3.43, 3.50)	Ref	Ref	Ref
	25–34 years	3.59(3.56, 3.62)	0.13(0.09, 0.17)	0.13(0.09, 0.18)	0.14(0.08, 0.19)
	35–44 years	3.65(3.62, 3.68)	0.19(0.15, 0.24)	0.20(0.15, 0.24)	0.19(0.14, 0.25)
	45+ years	3.58(3.56, 3.61)	0.12(0.08, 0.17)	0.13(0.08, 0.17)	0.10(0.04, 0.15)
People considering suicide often send out warning signs	15–24 years	3.15(3.11, 3.19)	Ref	Ref	Ref
	25–34 years	3.31(3.28, 3.33)	0.16(0.12, 0.20)	0.16(0.12, 0.20)	0.18(0.13, 0.23)
	35–44 years	3.38(3.35, 3.41)	0.22(0.18, 0.27)	0.23(0.18, 0.28)	0.24(0.17, 0.29)
	45+ years	3.41(3.38, 3.44)	0.26(0.22, 0.31)	0.27(0.22, 0.31)	0.28(0.23, 0.34)
Poor mental health is a workplace health and safety issue	15–24 years	4.21(4.18, 4.23)	Ref	Ref	Ref
	25–34 years	4.23(4.21, 4.25)	0.02(−0.01, 0.06)	0.02(−0.01, 0.05)	0.02(−0.01, 0.06)
	35–44 years	4.12(4.10, 4.15)	−0.08(−0.12, −0.04)	−0.09(−0.13, −0.05)	−0.09(−0.14, −0.05)
	45+ years	4.09(4.07, 4.11)	−0.12(−0.15, −0.08)	−0.12(−0.16, −0.09)	−0.14(−0.19, −0.10)
The construction industry must do something to reduce suicide rates	15–24 years	4.24(4.22,4.27)	Ref	Ref	Ref
	25–34 years	4.29(4.27, 4.31)	0.05(0.02, 0.08)	0.05(0.02, 0.08)	0.04(0.01, 0.08)
	35–44 years	4.25(4.23, 4.27)	0.01(−0.03, 0.04)	0.00(−0.03, 0.04)	−0.02(−0.06, 0.02)
	45+ years	4.25(4.22, 4.27)	0.00(−0.03, 0.03)	−0.00(−0.03, 0.03)	−0.02(−0.06, 0.02)

Model a: adjusted for state of training, year of training and training session. Model b: Model a + experience of knowing someone who had attempted/died by suicide. Model c: Model a + occupation.

**Table 3:** Predicted mean belief change and mean differences in belief change.

Belief	Age Group	Predicted Mean Belief Change *	Mean Difference in Belief Change, β Coefficient (95% CI)		
			Model a	Model b	Model c
Talking about suicide can cause suicide	15–24 years	0.00(−0.02, 0.03)	Ref	Ref	Ref
	25–34 years	0.01(−0.01, 0.03)	0.01(−0.03, 0.04)	0.01(−0.03, 0.04)	−0.02(−0.06, 0.02)
	35–44 years	−0.00(−0.03, 0.02)	−0.01(−0.05, 0.03)	−0.01(−0.05, 0.03)	−0.05(−0.10, −0.01)
	45+ years	−0.00(−0.03, 0.02)	−0.01(−0.04, 0.03)	−0.01(−0.04, 0.03)	−0.04(−0.09, 0.01)
People considering suicide often send out warning signs	15–24 years	0.47(0.43, 0.50)	Ref	Ref	Ref
	25–34 years	0.46(0.43, 0.48)	−0.01(−0.05, 0.03)	−0.01(−0.05, 0.03)	−0.05(−0.10, −0.00)
	35–44 years	0.40(0.37, 0.42)	−0.07(−0.11, −0.03)	−0.07(−0.11, −0.03)	−0.10(−0.15, −0.05)
	45+ years	0.38(0.36, 0.41)	−0.08(−0.13, −0.04)	−0.09(−0.13, −0.04)	−0.13(−0.18, −0.08)
Poor mental health is a workplace health and safety issue	15–24 years	0.16(0.13, 0.18)	Ref	Ref	Ref
	25–34 years	0.16(0.14, 0.18)	−0.00(−0.03, 0.03)	0.00(−0.03, 0.03)	−0.01(−0.05, 0.02)
	35–44 years	0.15(0.13, 0.17)	−0.01(−0.04, 0.02)	−0.01(−0.04, 0.02)	−0.03(−0.07, 0.01)
	45+ years	0.13(0.11, 0.15)	−0.03(−0.06, 0.00)	−0.02(−0.05, 0.01)	−0.04(−0.08, −0.00)
The construction industry must do something to reduce suicide rates	15–24 years	0.17(0.15, 0.20)	Ref	Ref	Ref
	25–34 years	0.17(0.15, 0.19)	−0.00(−0.03, 0.02)	−0.00(−0.03, 0.02)	−0.03(−0.06, 0.01)
	35–44 years	0.14(0.12, 0.15)	−0.04(−0.07, −0.01)	−0.04(−0.07, −0.01)	−0.06(−0.09, −0.02)
	45+ years	0.12(0.10, 0.14)	−0.06(−0.09, −0.03)	−0.06(−0.09, −0.03)	−0.09(−0.12, −0.06)

Model a: adjusted for state of training, year of training and training session. Model b: Model a + experience of knowing someone who had attempted/died by suicide. Model c: Model a + occupation. * Higher scores for belief change indicate greater changes toward desirable/better beliefs.

## Supplementary Materials

The following are available online at http://www.mdpi.com/1660-4601/16/3/475/s1, Table S1: Occupational differences in baseline beliefs by age groups.
