# Supplementary material for: Are Young Men Getting the Message? Age Differences in Suicide Prevention Literacy among Male Construction Workers
Source: Int J Environ Res Public Health. 2019 Feb 6;16(3):475. doi: 10.3390/ijerph16030475 (PMC6388151; doi:10.3390/ijerph16030475)
Supplement: Supplementary file 1 [file ijerph-16-00475-s001.pdf]

Table S1. Occupational differences in baseline beliefs by age groups: predictive margins for pre-test beliefs (also appearing in Figure 1).

	Occupational Group	Age Group			
		15-24 years	25-34 years	35-44 years	45+ years
Talking about suicide can cause suicide	Managers	3.61(3.55,3.67)	3.75(3.70,3.80)	3.80(3.75,3.86)	3.71(3.66,3.76)
	Professionals	3.72(3.60,3.83)	3.85(3.74,3.97)	3.91(3.79,4.02)	3.81(3.70,3.93)
	Technicians & Trades-workers	3.49(3.44,3.54)	3.63(3.59,3.66)	3.69(3.64,3.73)	3.59(3.54,3.64)
	Clerical & Administrative Workers	3.59(3.43,3.76)	3.73(3.57,3.89)	3.78(3.63,3.94)	3.69(3.53,3.85)
	Machinery Operators & Drivers	3.37(3.30,3.44)	3.51(3.45,3.56)	3.56(3.51,3.62)	3.47(3.41,3.52)
	Labourers	3.41(3.36,3.46)	3.55(3.50,3.59)	3.60(3.56,3.65)	3.51(3.46,3.56)
People considering suicide often send out warning signs or invitations	Managers	3.20(3.14,3.27)	3.38(3.33,3.44)	3.44(3.38,3.50)	3.48(3.43,3.54)
	Professionals	3.34(3.20,3.48)	3.52(3.38,3.66)	3.58(3.44,3.72)	3.63(3.49,3.76)
	Technicians & Trades-workers	3.12(3.07,3.17)	3.30(3.26,3.34)	3.35(3.31,3.40)	3.40(3.35,3.45)
	Clerical & Administrative Workers	3.30(3.12,3.48)	3.48(3.31,3.66)	3.54(3.36,3.71)	3.58(3.41,3.76)
	Machinery Operators & Drivers	3.05(2.98,3.11)	3.23(3.17,3.29)	3.28(3.23,3.34)	3.33(3.28,3.38)
	Labourers	3.15(3.10,3.20)	3.33(3.29,3.38)	3.39(3.34,3.44)	3.43(3.38,3.48)
Poor mental health is a workplace health and safety issue	Managers	4.37(4.32,4.42)	4.39(4.35,4.44)	4.28(4.23,4.32)	4.23(4.19,4.27)
	Professionals	4.41(4.32,4.50)	4.43(4.34,4.52)	4.32(4.23,4.41)	4.27(4.18,4.36)
	Technicians & Trades-workers	4.19(4.15,4.23)	4.21(4.19,4.24)	4.10(4.06,4.13)	4.05(4.01,4.08)
	Clerical & Administrative Workers	4.20(4.05,4.35)	4.22(4.07,4.37)	4.11(3.96,4.26)	4.06(3.90,4.21)
	Machinery Operators & Drivers	4.13(4.08,4.18)	4.15(4.11,4.19)	4.03(3.99,4.08)	3.98(3.94,4.03)
	Labourers	4.21(4.17,4.25)	4.23(4.20,4.27)	4.12(4.08,4.16)	4.07(4.03,4.10)
The construction industry must do something to reduce suicide rates	Managers	4.40(4.36,4.45)	4.45(4.41,4.48)	4.38(4.35,4.42)	4.38(4.35,4.42)
	Professionals	4.43(4.34,4.53)	4.47(4.38,4.57)	4.41(4.32,4.51)	4.41(4.32,4.50)
	Technicians & Trades-workers	4.26(4.22,4.30)	4.30(4.27,4.33)	4.24(4.21,4.27)	4.24(4.21,4.27)
	Clerical & Administrative Workers	4.43(4.31,4.54)	4.47(4.36,4.58)	4.41(4.29,4.52)	4.41(4.29,4.52)
	Machinery Operators & Drivers	4.15(4.09,4.20)	4.19(4.14,4.23)	4.13(4.08,4.17)	4.12(4.08,4.17)
	Labourers	4.23(4.19,4.27)	4.27(4.24,4.31)	4.21(4.17,4.25)	4.21(4.17,4.25)
